# Caregiver adverse childhood experiences and burden: associations with child welfare risk in a caregiver-child-sample

**DOI:** 10.3389/frcha.2026.1827054

**Published:** 2026-07-15

**Authors:** Fabienne Albert, Julia Franziska Baschab, Justine Hussong, Dominik Leininger, Eva Möhler, Katja Kauczor-Rieck

**Affiliations:** 1 Department of Child and Adolescent Psychiatry, Saarland University, Homburg, Germany; 2 Child and Adolescent Psychiatry, Saarland University Medical Center, Homburg, Germany

**Keywords:** adverse childhood experiences (ACE), caregiver burden, child abuse potential, child welfare risk, intergenerational transmission of trauma

## Abstract

**Background:**

Adverse Childhood Experiences (ACEs) are well-established risk factors for long-term psychosocial impairments and can affect both parental functioning and child well-being. The present study investigates the impact of caregiver ACEs on child welfare risk over the course of a caregiver-focused intervention.

**Methods:**

41 caregiver-child dyads (children: *M* = 4.85 years, *SD* = 1.41) were assessed at three time points: baseline (T1), after 4 weeks of intervention (T2), and six months post (T3). Caregiver ACEs were measured using a standardized questionnaire (ACE), and child welfare risk was assessed with a short form of the German version of the Child Abuse Potential Inventory (CAPI) [Eltern-Belastungs-Screening zur Kindeswohlgefährdung (EBSK)]. Linear mixed-effects models (LMMs) examined changes in EBSK scores over time and the influence of ACE number and domain.

**Results:**

Child welfare risk did not decrease immediately post-treatment (T1–T2), but a significant reduction was observed at follow-up (T2–T3). Higher caregiver ACE scores were consistently associated with elevated EBSK scores. Analyses of ACE domains revealed that history of parental household dysfunction, but not abuse, significantly predicted higher child welfare risk. The number of ACEs (≥4 vs. <4) did not significantly moderate EBSK trajectories.

**Conclusions:**

Child welfare risk scores were lower at follow-up than at post-treatment assessment. However, given the absence of a control group, these findings should be interpreted as temporal associations rather than evidence of intervention effects. Caregiver ACEs, particularly history of household dysfunction, remain a persistent risk factor for child welfare. Systematic assessment of ACEs can help identify at-risk families and implement trauma-informed interventions, follow-up support, and professional training in clinical settings.

## 1 Introduction

Children with behavioral problems or mental disorders often grow up in families characterized by high parental stress and are at an increased risk of experiencing child maltreatment (1, 45). Therefore, the early identification of parental stress and the provision of targeted support in parenting represent essential preventive strategies to reduce overload and prevent negative developmental trajectories (1). Early childhood is considered a particularly sensitive and vulnerable developmental period (2). During this time, fundamental attachment patterns, learning abilities, emotional regulation skills, and social competencies are formed, and early childhood experiences are closely linked to later mental disorders (2, 3). Interventions and support during this stage therefore represent highly sustainable preventive measures that promote long-term mental health (2, 4). The close association between maternal psychological distress and child development becomes evident already during pregnancy (5). High levels of maternal stress influence hormonal processes, which in turn affect gene expression in the fetal brain and the infant's later stress regulation capacity. As a result, a vicious cycle may emerge early on: a psychologically burdened mother interacts with a highly reactive and easily stressed infant, increasing the likelihood of dysfunctional interaction patterns (6, 7). Adverse Childhood Experiences (ACEs), including abuse, neglect, and household dysfunction, are well-documented risk factors for long-term psychological, somatic, and social impairments (8, 9). ACEs encompass both direct forms of maltreatment and exposure to dysfunctional household environments, such as parental substance abuse, mental illness, domestic violence, or criminal behavior. Large-scale epidemiological studies have demonstrated a strong dose–response relationship between the number of ACEs and adverse health outcomes across the lifespan (8–10). In a representative German sample, 43.7% of participants reported at least one adverse childhood experience, and 8.9% reported four or more ACEs; this high-risk group showed significantly elevated risks for depression, anxiety, physical aggression, and reduced life satisfaction (4, 11). Prevalence rates of specific forms of maltreatment in Germany further underline the public health relevance of early adversity (12). Beyond their direct long-term consequences, ACEs are increasingly examined within an intergenerational framework (4).

Research suggests that caregivers with a history of childhood adversity often exhibit heightened stress reactivity, emotional dysregulation, and impaired relational patterns, which may negatively affect their parenting behavior and their children's psychological well-being (13, 14). Parental burden is widely recognized as a central predictor of child developmental trajectories and potential child welfare risk (45). Unresolved trauma and chronic stress reduce parental coping capacity and increase vulnerability to parenting stress, a finding supported by further research (13, 15, 16). While Lange et al. (13) identified a significant association between the number of maternal ACEs and parental stress, they did not find a direct link with specific parenting behaviors. In contrast, a study of young mothers reported that three or more ACEs were significantly associated with maternal psychological symptoms, increased parenting stress, and both internalizing and externalizing symptoms in children (14). A parental ACE score of ≥4 was associated with a significantly increased risk of behavioral difficulties in children, with maternal ACEs showing a particularly strong influence (17). Altpeter et al. (18) reported a higher ACE -load in parents of child psychiatric patients compared with community samples. Also, infants with symptoms of regulatory and other mental disorders have been reported to have caregivers with higher ACE-Scores than the general population. These findings suggest that parental burden may represent a key mediating mechanism linking caregiver ACEs to adverse child outcomes. However, although the long-term effects of ACEs are well established, comparatively few studies have investigated how caregivers' ACE histories relate to changes in child welfare risk over time, particularly within the context of clinical interventions (19). Addressing this gap is essential for developing targeted preventive and therapeutic strategies aimed at reducing parental stress, strengthening parenting capacities, and ultimately improving child mental health outcomes. Importantly these intergenerational processes are likely shaped by a complex interplay of genetic predispositions, environmental influences, and gene-environment interactions, which may moderate both risk transmission and treatment responsiveness (20, 21).

### 1.1 The present study

The present paper examines the course and potential influencing factors of risk for child welfare endangerment within an interaction-based inpatient parent-child treatment setting. It is part of a larger study evaluating the effectiveness of the treatment using the Emotional Availability (EA) Scales and specifically investigates whether the risk of potential child welfare endangerment changes over the course of treatment. In addition, the study analyzes the association between caregivers' adverse childhood experiences (ACEs) and the quality of caregiver-child interaction across different measurement time points. Finally, it examines whether caregivers in the inpatient parent-child unit report significantly higher ACE scores compared to the general population. Based on these research questions, the following hypotheses are proposed:

#### 1.1.1 H1: change in child welfare risk

- **H1a**: After 4 weeks of treatment (T2), child welfare risk scores are expected to be significantly lower than at treatment onset (T1).
- **H1b**: Child welfare risk scores are expected to remain at a stable, low level at the 6-month follow-up (T3), without further significant reduction.
- **H1c** (exploratory): The number of caregiver ACEs is expected to moderate the change in child welfare risk scores over time, such that caregiver with higher ACE scores show different EBSK trajectories compared to those with lower ACE scores.

#### 1.1.2 H2: influence of number and domain of ACEs on child welfare risk (exploratory)

- **H2a**: Caregivers with ≥4 ACEs are expected to report higher child welfare risk scores across the treatment course than caregivers with <4 ACEs.
- **H2b**: Specific ACE domains (e.g., abuse, household dysfunction) are expected to predict child welfare risk scores across the treatment course.

## 2 Method

The study evaluated treatment in the parent-child inpatient unit of the Clinic for Child and Adolescent Psychiatry, Psychosomatics and Psychotherapy at Saarland University Medical Center. The study was approved by the Ethics Committee of the Medical Association of Saarland (Ärztekammer des Saarlandes) and registered in the German Clinical Trials Register (DRKS). Written informed consent was obtained from all caregivers. Only the measures relevant to the present paper are described below.

### 2.1 Participants

The study included children aged 0–8 years referred to the child and adolescent psychiatric clinic for severe behavioral disorders with a child psychiatric diagnosis and their caregivers who received inpatient treatment on the parent–child ward between May 6, 2024, and February 12, 2026, provided that informed consent was obtained. Participation required basic German language skills. For statistical analyses, only participants who had completed at least four weeks of treatment were considered, resulting in a final sample of *N* = 41. No *a priori* sample size calculation was conducted, as the sample size was determined during the recruitment period.

### 2.2 Measures

**Sociodemographics.** Various demographic and background variables were assessed for children and caregivers (e.g., age, sex, treatment duration, waiting time, caregiver educational attainment, caregiver occupation). **Adverse childhood experiences**. Caregiver ACEs were measured using the Adverse Childhood Experiences Questionnaire (8, 22). The 10-item questionnaire retrospectively assesses experiences of abuse, household dysfunction, and neglect. Completion time is approximately 5 min. **Child welfare risk**. The Eltern-Belastungs-Screnning zur Kindeswohlgefährdung (EBSK (23); is a validated caregiver-report instrument assessing risk of future child maltreatment and neglect. It is a shortened German version of the Child Abuse Potential Inventory (CAPI (24); (CAPI). Completion time is approximately 5–10 min. The **EBSK** is a standardized questionnaire used to assess the risk of future physical child abuse and child neglect. It consists of a **burden scale** measuring the level of parental stress and burden, as well as **three validity scales** assessing social desirability, random responding, and inconsistent responding. The questionnaire takes approximately 5–10 min to complete. The instrument demonstrates high internal consistency (α = .91) and has been shown to possess good validity (23). Scores on the burden scale are interpreted as probability statements: higher scores indicate a greater likelihood of substantial parental burden and an increased risk of child welfare endangerment, whereas lower scores suggest that the respondent is exposed to relatively low levels of burden. The following cut-off values apply to the burden scale: <**161 points:** no indication of significant burden (unremarkable range), **161–184 points:** low level of burden, **185–206 points:** relatively high level of burden, **≥207 points:** very high level of burden. The EBSK is a screening instrument and should not be considered a substitute for a comprehensive clinical or diagnostic assessment.

#### 2.2.1 Intervention

At the Parent-Child Unit of the Saarland University Medical Center, children from birth to eight years of age primarily received inpatient treatment for early childhood mental health disorders, developmental delays, of significant behavioral difficulties. Older children were admitted if parent-child interaction difficulties were the primary concern. Therapeutic work was delivered within an interdisciplinary, psychotherapeutic framework using a video interaction-focused parent-child approach. Core components included:

- Video-supported interaction work: Recorded play sequences were analyzed to evaluate caregiver-child interactions and facilitate joint reflection in therapy (25).
- Caregiver group sessions and individual consultations: These sessions conveyed theoretical knowledge and practical skills related to secure attachment, core dimensions of emotional availability (sensitivity, structuring, non-hostility, non-intrusiveness), children's emotional needs, and strategies for managing challenging everyday situations (25–28).
- Biographical reflection and self-regulation: Caregivers were supported in reflecting on their own attachment histories and current stressors, and trained in self-care and emotion regulation strategies (29, 30).

The special parent-child-unit provides four treatment places for children admitted together with their primary caregivers within a general child and adolescent psychiatric hospital. The structured intervention was delivered by a multiprofessional team following standardized procedures to ensure consistent implementation. The unit provided four treatment places for children admitted together with their primary caregivers. The intervention was delivered by a multiprofessional team following standardized procedures to ensure consistent implementation. The minimum treatment duration was four weeks. The program included psychotherapeutic interventions for the children, regular parent counseling sessions, and one video-based interaction session per week.

### 2.3 Procedure

Families were informed about the study during the initial diagnostic consultation and invited to participate. Data collection occurred at three time points:

- T1 (baseline/inpatient admission): Caregivers completed the EBSK questionnaire.
- T2 (4 weeks after admission). Caregivers completed EBSK and ACE questionnaires.
- T3 (6-month follow-up): Caregivers completed the EBSK questionnaire.

### 2.4 Data analysis

Statistical analyses were conducted in R [Version 4.5.2 (31)]. The dataset was screened for missing values and univariate outliers [±3 *SD*; (32)]. Scale scores were computed when ≥80% of items were available (33). Two extreme values (one caregiver age: 62, one ACE score: 9) were retained; sensitivity analyses indicated they did not substantially affect results. Missing data were most pronounced at follow-up (T3), especially for EBSK scores (23/41 missing), while other variables were largely complete. Continuous predictors were grand-mean-centered. H1 was tested using linear mixed-effects models [LMMs, lme4, Version 3.1–3 (34)], with child emotional burden (EBSK) as the dependent variable, repeated measures nested within participants, and a random intercept for each child. Time was modeled using two contrasts, and ACE scores were included as fixed effects; child age was added in extended models. Models were estimated using maximum likelihood (REML = FALSE) and compared to nested and null models via likelihood ratio tests. Model assumptions were evaluated using residual plots, Q-Q plots, and variance inflation factors. H2 followed the same approach, replacing continuous ACE scores with ACE groups (H2a: <4 vs. ≥4) or domains (H2b: abuse vs. household dysfunction). Main models included ACE group/domain, with additional models adjusting for child age. Model comparisons and diagnostics were conducted analogously to H1.

### 2.5 Risk of bias

Several potential sources of bias should be considered. Only H1a and H1b were preregistered, while subsequent analyses were exploratory. The intervention and assessments were conducted within the same clinical setting, potentially introducing experimenter bias. However, treatment followed standardized procedures and was delivered by trained multiprofessional staff. Key variables (EBSK, ACE) relied on caregiver self-report, raising the possibility of shared method variance, although both are validated instruments. The sample was small with unequal ACE group sizes, limiting statistical power. Attrition at follow-up was substantial, but mixed-effects models allowed inclusion of incomplete cases under maximum likelihood estimation. Finally, the inpatient clinical setting may restrict generalizability while enhancing ecological validity for high-risk families.

## 3 Results

### 3.1 Descriptive statistics

**Figure 1:**
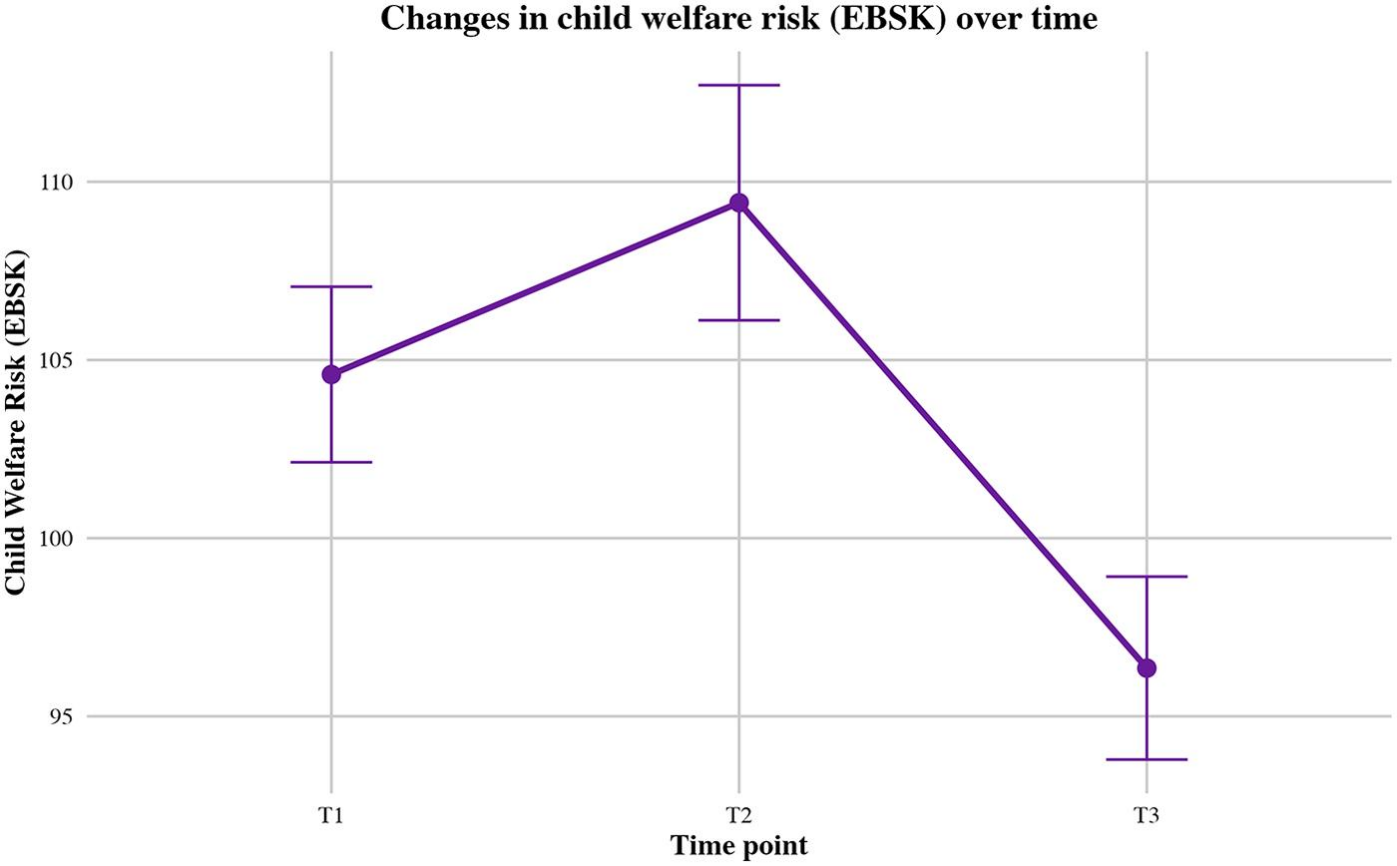
Mean EBSK rating of all caregivers over time. Error bars represent ± standard error.

The total sample comprised 41 caregivers and their children with a mean age of 4.85 (*SD* = 1.41, range = 1–8). Most children were male (70.7%). Primary caregivers had a mean age of 35.34 years (*SD* = 6.20, range = 25–62). Regarding educational attainment, nearly half of the caregivers held an intermediate secondary school certificate, while approximately one third had obtained a higher education entrance qualification. The mean ACE score of caregivers was 2.25 (*SD* = 2.15, range = 0–9). Caregiver-reported risk of child well-being as assessed by the EBSK indicated moderate to high levels at baseline, with variation across measurement points. Mean treatment duration was 46.11 weeks (*SD* = 11.53, range = 25–80). Descriptives statistics of demographic and clinical characteristics are presented in Table 1 and Figure 1.

**Table 1:** Demographic and clinical characteristics of the total sample (*N* = 41).

Variable	
Age of child (years), *M* (*SD*)	4.85 (1.41)
Sex of child	
Female, *n* (%)	12 (29.3%)
Male, *n* (%)	29 (70.7%)
Age of caregiver (years), *M* (*SD*)	35.34 (6.20)
Sex of caregiver	
Female, *n* (%)	37 (90.2%)
Male, *n* (%)	4 (9.8%)
Education level of caregiver	
No school qualification, *n* (%)	2 (4.9%)
Lower secondary school/special education, *n* (%)	7 (17.1%)
Intermediate secondary school certificate, *n* (%)	20 (48.8%)
Higher education entrance qualification, *n* (%)	12 (29.3%)
ACE score, *M* (*SD*)	2.25 (2.15)
EBSK burden	
T1, *M* (*SD*)	104.59 (15.78)
T2, *M* (*SD*)	109.41 (21.13)
T3, *M* (*SD*)	96.35 (16.44)

EBSK = child welfare risk, measured with the Eltern-Belastungs-Screnning zur Kindeswohlgefährdung [Parent Stress Screening for Risk to Child Well-Being; (22)]. ACE, Adverse Childhood Experiences, measured with the ACE-D questionnaire (23).

To examine potential attrition bias, T3 completers (*n* = 18) and non-completers (*n* = 23) were compared on baseline characteristics. No significant differences were found for baseline child welfare risk at T1 (*p* = .552, *d* = 0.19) or child age (*p* = .333, *d* = 0.31). For caregiver adverse childhood experiences (ACEs), a trend-level difference was observed, with higher ACE scores among non-completers (*p* = .077, *d* = 0.56). Overall, these findings do not provide evidence for systematic attrition bias in baseline characteristics, although the observed effect for caregiver ACE scores suggests a potential trend that should be interpreted cautiously given the limited sample size and corresponding statistical power.

### 3.2 H1: change in child welfare risk (EBSK)

To examine changes in child welfare risk over time, linear mixed-effects models with random intercepts for children were fitted. Time was modeled using two planned contrasts: C1 (change from T1 to T2, reflecting change after treatment) and C2 (change from T2 to T3, reflecting change during follow-up). Models were estimated using maximum likelihood, and significance testing was based on Satterthwaite's approximation for degrees of freedom. Model assumptions were met; residuals were approximately normally distributed, homoscedasticity was satisfied, no influential cases were identified, and multicollinearity was low (all VIFs < 2). First, a model including only the planned time contrasts provided a significantly better fit to the data than the null model, [*χ*^2^(2) = 8.16, *p* = .017], indicating overall change in child welfare risk across measurement occasions. While no significant change was observed from T1 to T2 (C1: b = −1.21, *p* = .550), child welfare risk decreased significantly from T2 to T3 (C2: b = −6.69, *p* = .011). In a second step, caregiver adverse childhood experiences (ACEs; grand-mean centered) were added to the model to examine whether they explained variance in child welfare risk beyond time. The inclusion of ACEs significantly improved model fit compared to the time-only model, *χ*^2^(1) = 6.24, *p* = .013. As shown in Table 2, higher caregiver ACEs were associated with higher levels of child welfare risk across all measurement occasions (*b* = 2.68, *p* = .013). The pattern of the time contrasts remained unchanged in this extended model. Adding an interaction between time and ACEs did not significantly improve model fit, *χ*^2^(2) = 1.56, *p* = .460, indicating that the association between caregiver ACEs and child welfare risk did not differ across time. Similarly, including child age as a covariate did not lead to a significant improvement in model fit, *χ*^2^(1) = 0.97, *p* = .325. Therefore, the more parsimonious model including time contrasts and caregiver ACEs was retained as the final model. This model explained 15.5% of the variance through fixed effects (marginal *R*^2^) and 46.1% of the total variance (conditional *R*^2^).

**Table 2:** Linear mixed-effects model predicting child welfare risk over time (H1).

Predictor	b	*SE*	95% CI	*t-* value	*p*
C1	−0.98	1.99	[−4.93, 2.99]	−0.50	.623
C2	−6.13	2.53	[−11.16, −1.10]	−2.42	.018*
ACE	2.68	1.03	[0.62, 4.75]	2.60	.013*

b = unstandardized regression coefficient; *SE*, standard error; 95% CI, 95% confidence interval for b. C1 = planned contrast for change from T1 to T2 (H1a), C2 = planned contrast for change from T2 to T3 (H1b). ACE, caregiver adverse childhood experiences, grand-mean centered.

* *p* < .05.

### 3.3 H2: influence of number and type of ACEs on child welfare risk (exploratory)

Linear mixed-effects models were fitted to examine the influence of ACE group (H2a: ≥4 vs. <4 ACEs) and specific ACE domains (H2b: abuse vs. household dysfunction) on child welfare risk over time. Time was modeled using the same planned contrasts as in H1: C1 (T1:T2) and C2 (T2:T3) and random intercepts for children were included. Model assumptions were met, with approximately normally distributed residuals, homoscedasticity, and no influential data points detected.

#### 3.3.1 H2a: influence of number of ACEs on child welfare risk (EBSK; exploratory)

Of the 41 caregivers, *n* = 9 (22.0%) reported four or more ACEs, whereas *n* = 32 (78.0%) reported fewer than four ACEs. The model including C1, C2, and ACE group provided a significantly better fit than the null model, [*χ*^2^(3) = 10.03, *p* = .018]. Likelihood ratio tests indicated that adding an interaction between time and ACE group did not improve model fit, *χ*^2^(2) = 2.09, *p* = .351, nor did including child age as a covariate, *χ*^2^(1) = 1.66, *p* = .198. As shown in Table 3, child welfare risk changed across measurement occasions in the same pattern as observed in H1. The main effect of ACE group (≥4 ACEs) was not significant (b = 7.72, *p* = .166). The model explained 9.2% of variance through fixed effects (marginal *R*^2^) and 45.3% overall (conditional *R*^2^). However, because only nine caregivers were classified into the high-ACE group (≥4 ACEs), the analysis was likely underpowered to detect effects of plausible magnitude. Consequently, the non-significant finding should be interpreted as inconclusive rather than as evidence that caregiver ACEs are unrelated to child welfare risk.

**Table 3:** Linear mixed-effects model predicting child welfare risk over time (H2a; number of ACEs).

Predictor	b	*SE*	95% CI	*t*-value	*p*
C1	−1.08	2.00	[−5.06, 2.90]	−0.54	.590
C2	−6.41	2.55	[−11.49, −1.35]	−2.52	.014*
Ace ≥ 4	7.72	5.48	[−3.24, 18.74]	1.41	.166

b = unstandardized regression coefficient; *SE*, standard error; 95% CI, 95% confidence interval for b. C1 = planned contrast for change from T1 to T2, C2 = planned contrast for change from T2 to T3. ACE group ≥ 4 = caregivers with four or more ACEs. Time contrasts: T1 = baseline, T2 = post-treatment, T3 = follow-up.

* *p* < .05.

#### 3.3.2 H2b: influence of specific ACE domains on child welfare risk (EBSK; exploratory)

Across caregivers, the mean number of abuse-related ACEs was 1.05 (*SD* = 1.24), and the mean number of household dysfunction ACEs was 1.15 (*SD* = 1.24). The model including C1, C2, ACE Abuse, and ACE HHD significantly improved fit compared to the null model, *χ*^2^(4) = 16.75, *p* = .002. Adding interactions between time and either ACE domain did not improve model fit, *χ*^2^(4) = 1.61, *p* = .806, nor did including child age as a covariate, *χ*^2^(1) = 0.26, *p* = .609. As shown in Table 4, child welfare risk decreased over time in a pattern similar to H1. Regarding ACE domains, ACE Abuse was not associated with child welfare risk (*b* = −0.01, *p* = .996), whereas ACE HHD was significantly associated with higher child welfare risk across all time points (*b* = 5.38, *p* = .009). The model explained 18.7% of the variance through fixed effects (marginal *R*^2^) and 46.3% overall (conditional *R*^2^). These findings suggest that household dysfunction, but not abuse, contributes to higher child welfare risk, independent of changes over time.

**Table 4:** Linear mixed-effects model predicting child welfare risk over time (H2b: specific ACE domains).

Predictor	b	*SE*	95% CI	*t-* value	*p*
Time C1	−1.04	1.98	[−4.98, 2.92]	−0.52	.602
Time C2	−6.25	2.52	[−11.26, −1.24]	−2.48	.016*
ACE Abuse	−0.01	1.95	[−3.92, 3.90]	−0.01	.996
ACE HHD	5.38	1.96	[1.46, 9.33]	2.74	.009**

b = unstandardized regression coefficient; *SE*, standard error; 95% CI, 95% confidence interval. C1 = planned contrast for change from T1 to T2, C2 = planned contrast for change from T2 to T3. ACE Abuse = caregiver exposure to abuse, grand-mean centered; ACE HHD = caregiver household dysfunction, grand-mean centered. Time contrasts: T1 = baseline, T2 = post-treatment, T3 = follow-up.

* *p* < .05.

** *p* < .01.

## 4 Discussion

### 4.1 Summary of results

The present study examined changes in child welfare risk (EBSK) over the course of a caregiver-focused intervention and explored the influence of caregivers' adverse childhood experiences (ACEs) on these changes.

### 4.2 H1: change in child welfare risk over time

Child welfare risk did not significantly decrease immediately after treatment (T1 to T2), but a significant reduction was observed from post-treatment to follow-up (T2 to T3). Higher caregiver ACEs were associated with higher EBSK scores across all time points. These results indicate that lower EBSK scores were observed at follow-up compared to post-treatment assessment. However, because no control group was included, it remains unclear whether this pattern reflects intervention-related change, natural developmental processes, regression to the mean, or other unmeasured influences.

### 4.3 H2: influence of number and domain of ACEs on child welfare risk

Given that only nine caregivers were classified into the high-ACE group, the analysis was likely underpowered to detect effects of plausible magnitude. Therefore, this finding should be interpreted as inconclusive rather than as evidence for the absence of an effect. Analyses of domain-specific ACEs revealed that caregiver exposure to household dysfunction (HHD), but not abuse, was significantly associated with higher child welfare risk across all time points. No significant interactions with time were observed for either ACE domain or ACE group, indicating that the temporal pattern of child welfare risk reduction was independent of caregiver ACE exposure. Altogether, these findings indicate that child welfare risk tends to decrease over the intervention period, with effects becoming more pronounced at follow-up. Caregiver ACEs, particularly household dysfunction, are consistently associated with higher child risk, whereas the number of ACEs alone or exposure to abuse did not significantly alter the trajectory.

### 4.4 Interpretation and theoretical implications

The present findings confirm the hypothesis that higher parental ACE scores are associated with increased parental stress and a higher risk of child welfare endangerment. Caregivers with higher ACE levels reported elevated child welfare risk scores. Furthermore, caregivers admitted to our parent-infant-clinic report a higher ACE-Load than the german general population in accordance with reports for other child psychiatric populations (18). These findings support the concept of intergenerational transmission of stress and trauma (13, 35, 36) and align with theoretical models proposed by Lyons-Ruth and Block (7), which suggest that early internalized relational experiences and maladaptive emotion regulation strategies may be reactivated in the context of parenting, contributing to increased psychosocial burden. The results further suggest that chronic familial stress can exert a persistent influence on child well-being Hughes et al. (9). Meta-analytic evidence indicates that parental ACEs, in general, are associated with increased psychological distress and a higher risk of suboptimal child outcomes (37). In particular, cumulative exposure (ACEs ≥ 4) substantially increases the likelihood of psychological difficulties in general. In the present study, household dysfunction emerged as particularly influential factor. Parents with such experiences often have a history of prolonged chronic insecurity, low sensitivity, and unstable caregiving, which can impair their own attachment security and emotion regulation, thereby increasing the risk for own psychopathology (38, 39, 46). Consequently, difficulties in interacting with their own child may arise, as reflected in elevated EBSK scores. An unexpected finding in the present study is that parental abuse experiences do not influence the welfare risk of the child. One explanatory approach may differentiate between single traumatic events (e.g., domestic abuse as a potential mono-trauma) and chronic family stressors (40). While acute experiences of violence may be associated with classic post-traumatic stress responses, chronic family adversities are more strongly linked to attachment-related trauma and complex adaptation processes. Chronic dysfunctional family conditions are often less clearly delineated for affected parents and may be internalized as “normal”, making reflection on maladaptive patterns more difficult (8, 41). Parents with experiences of domestic abuse may be more motivated to consciously break intergenerational cycles, whereas chronic household dysfunction can exert a more subtle, long-term influence on parenting practices (4). However, different reports in a larger sample have shown an impact of parental history of abuse on emotional availability in the parent child-context (42) with a small but significant effect size, indicating that the present sample might by too small to detect a potential relationship between parental abuse and child welfare. The observed temporal pattern is compatible with the assumption that interaction-focused interventions may contribute to reductions in child welfare risk. However, the present design does not allow causal attribution of these changes to the intervention itself. The observed increase in EBSK scores between T1 and T2 may not necessarily indicate an actual deterioration in caregiving capacities. Rather, treatment may have enhanced caregivers' awareness of their own stress, emotional responses, and relational difficulties, leading to a more critical self-assessment during the intervention process (Fonagy et al. 2015). Additionally, the observed changes between T2 and T3 could be explained by a gradual shift in focus toward the child's needs. At the beginning of the intervention, caregivers' attention to their child's emotional and psychological needs may have been limited. Over the course of treatment, however, a sustained change in interactions with the child and greater sensitivity to their needs appears to have occurred. In this context, caregiver attachment security plays a central role. A secure attachment representation is considered a protective factor for successful interactions, positive parenting attitudes, and the mental health of both parents and children (46). Subjective parental stress is closely related to emotion regulation and attachment organization (43). A key treatment goal of attachment-based interventions is therefore to enhance caregivers' attachment security (44). Since changes in internal working models and emotion regulation strategies require time, stronger effects observed from T2 to T3 are plausible (Fonagy et al. 2015). Overall, these findings underscore the importance of attachment-oriented, interaction-focused interventions that enhance both parental emotion regulation and sensitivity to the child's needs. The delayed effects between T2 and T3 suggest that sustainable changes in internal working models and interaction patterns require a longer developmental process. At the same time, it must be considered that observed changes may have been influenced by external factors or normative developmental processes, as the study lacked a control group and relevant covariates. Therefore, causal interpretations are not possible.

### 4.5 Practical and clinical implications

Our findings have important implications for child and adolescent mental health services. Early screening for caregiver ACE exposure could help identify families who may benefit from additional support, particularly when child psychopathology is present. Interaction-focused treatment programs can strengthen caregiver resources and well-being, and support the development of positive parent-child interactions. By improving interaction quality and addressing parental stress, such interventions not only benefit the child but also empower caregivers in their role, fostering constructive dyadic relationships and promoting healthy developmental outcomes.

### 4.6 Limitations, strengths and future studies

Despite its longitudinal design, several limitations should be acknowledged. The inpatient design without a control group precludes causal conclusions regarding intervention effects. ACEs were assessed retrospectively at a single time point with a screening tool and may be subject to recall bias or social desirability, potentially affecting reporting accuracy. In addition, unmeasured contextual factors (e.g., caregiver psychopathology or chronic environmental stress) may have influenced the observed associations. The modest sample size and exploratory components of the analyses increase the risk of both Type I and Type II errors. Attrition at follow-up further limits the robustness of longitudinal interferences, although delayed effects remained detectable despite reduced statistical power. Findings should therefore be interpreted cautiously. Notwithstanding these constraints, the study advances the field in several important ways. It embeds intergenerational ACE research within an attachment-oriented clinical intervention and applies a longitudinal follow-up design capable of detecting delayed change processes. By differentiating cumulative from domain-specific ACE exposure, the findings identify household dysfunction as a particularly salient pathway of intergenerational risk, thereby refining models of stress transmission and offering clinically actionable implications. An additional limitation concerns the timing of ACE assessment. Caregivers completed the ACE questionnaire at T2, after four weeks of treatment. Because the intervention included reflection on personal attachment histories and current stressors, participation may have increased the salience or accessibility of childhood memories. Consequently, retrospective ACE reporting could have been influenced by the intervention itself. For consistency with prior ACE research, ACE exposure was categorized using the commonly applied threshold of four or more ACEs. However, dichotomization may reduce statistical power and obscure potentially meaningful dose-response relationships. Continuous ACE analyses were therefore additionally conducted in H1 and revealed significant associations between ACE load and child welfare risk. Future research should employ larger, controlled, multi-site designs to strengthen causal inference and external validity. Incorporating multi-informant assessments and objective measures of parent–child interaction would reduce reliance on self-report, and extended follow-up could clarify the durability of intervention effects. Additionally, future work should investigate the mechanisms by which caregiver ACEs, particularly household dysfunction, influence child welfare risk, and explore the generalizability of these findings across different clinical and community settings.

### 4.7 Conclusion

Early, caregiver-inclusive interventions are critical for supporting mental health in infancy and preschool age. Multimodal, interaction-focused programs have been proposed to improve parent–child interaction quality, reduce caregiver stress, and foster positive developmental outcomes in children Caregivers' adverse childhood experiences (ACEs), particularly household dysfunction, are indicated to remain influential and highlight the need for systematic screening and trauma-informed, family-centered support (44). Child welfare indicators were lower at follow-up than at post-treatment assessment, although the present design does not permit conclusions regarding whether these changes were attributable to the intervention. Integrating parent-focused approaches, system-oriented strategies, and attention to child temperament can be hypothesized to further optimize outcomes. Expanding interventions to day-clinic formats or additional age groups might increase accessibility and sustainability while reducing the economic burden of full inpatient care. Overall, these findings underscore the importance of early identification, targeted support of parental biography child and adolescent psychiatric patients. Trauma-informed, family-centered interventions may support caregiver well-being and parent-child relationships and represent a promising approach for addressing intergenerational adversity. Further controlled studies are needed to evaluate their effectiveness.

## Data Availability

The raw data supporting the conclusions of this article will be made available by the authors, without undue reservation.
